# A miniature low-immunogenic platform for the biosynthesis of self-assembling protein nanoparticles

**DOI:** 10.7150/ntno.98946

**Published:** 2025-03-03

**Authors:** Almina I. Polinova, Anna V. Serkina, Marina V. Volkova, Aleksandr A. Gorbunov, Evgeniia P. Sannikova, Irek I. Gubaidullin, Aleksandr S. Komolov, Anna V. Rybakova, Marina Yu. Kopaeva, Konstantin S. Plokhikh, Georgy S. Peters, Artem A. Shatilov, Alexander A. Shtil, Galina A. Posypanova, Alexander P. Trashkov, Natalia V. Bulushova, Dmitry G. Kozlov

**Affiliations:** 1 National Research Center «Kurchatov Institute», Moscow, 123182, Russia.; 2 National Research Center «Kurchatov Institute» - GOSNIIGENETIKA, Kurchatov Genomic Center, Moscow, 117545, Russia.; 3 National Research Center Institute of Immunology Federal Medical-Biological Agency of Russia, Moscow, 1115522, Russia.; 4 Blokhin National Medical Research Center of Oncology, Moscow, 115522, Russia.

**Keywords:** self-assembling protein nanoparticle, self-associating peptide, artificial vaccine platform, subunit vaccine, immunogenicity, OVA_257-280_ antigen, N-terminal formylmethionine

## Abstract

**Aims:** Previously, to obtain antigen-presenting self-assembling protein nanoparticles (SAPN), we developed a biosynthetic platform combining the self-associating peptide L_6_KD and the SUMO protein. In the current work, the immunogenic SUMO was replaced with an artificial 30 amino acid long peptide pepA1.

**Methods:** The immunogenic properties of the pepA1-SAPN were tested in mice using the pneumococcal PhtD19 and ovalbumin OVA_257-280_ antigens in the absence of adjuvants.

**Results and Conclusions:** The updated SAPN showed a 100% seroconversion rate and low immunogenicity of the platform. Given the effective synthesis and improved purification procedure, the pepA1-based miniature platform looks promising for development of vaccines and vehicles for targeted delivery.

## Introduction

The challenges caused by the sudden emergence of the highly contagious human viral infection in the absence of an efficient vaccine clearly demonstrated that the development of vaccines against new pathogens needs to be boosted. In this regard, of particular interest is designing universal recombinant platforms with low self-immunogenicity, which can dramatically accelerate and cheapen the manufacturing process. In particular, one promising direction in vaccine engineering is antigen presentation on the surface of nanoparticles 1. The prolonged half-life of the nanoparticles and the grouped localization of antigen on their surface promotes the development of a robust immune response 2. In particular, of great interest are self-assembling protein nanoparticles (SAPN) 3, 4. They can be synthesized in bacterial systems, which is not only quicker and safer but more environmentally friendly than traditional methods 5. Previously, we created SAPN, the subunits of which represent fusion proteins, consisting of the L_6_KD-peptide, adaptor protein SUMO and target 6.

The self-assembling peptide (SAP) L_6_KD plays a key role in the three-component fusions. As a member of the surfactant-like peptide family, L_6_KD can spontaneously form nanoscale particles resistant to external influences 7-9.

Unlike capsid proteins from viruses, L_6_KD features a significantly simplified self-assembly mechanism, which enables better control over nanoparticle formation and composition. Notably, when synthesized as part of hybrid proteins, SAP L_6_KD facilitates their self-assembly 9-11.

SAP-containing fusion proteins were synthesized in inclusion bodies in *E. coli* cells. During the purification procedure, these proteins underwent renaturation and self-assembly into SAP nanoparticles (SAPNs). However, the ability of L_6_KD to drive SAPN formation had its limitations. Specifically, many short peptide fusions with this SAP could not be synthesized in *E. coli* without an adapter element. Additionally, the absence of an adapter likely resulted in steric hindrance that impaired the presentation of bulk antigens on the surface of SAPNs. Consequently, we believe that incorporating adapter elements significantly enhanced SAPN development by improving fusion biosynthesis and facilitating antigen presentation.

For the first-generation platform for SAPN development, we selected the SUMO protein of* Saccharomyces cerevisiae* as the adapter element 6, 12. SUMO's advantages include its small size, lack of cysteines, rapid and autonomous folding, distinct orientation of the N and C termini, high solubility, and its chaperone-like ability to aid in protein folding 13. Additionally, the availability of a SUMO-specific protease 14 enabled efficient and precise processing of SAPNs 6, 11.

Two adjuvant-free intraperitoneal injections of L_6_KD-SUMO based nanoparticles at 21-day intervals induced the secretion of IgG against the chosen peptide antigen in C56BL/6 mice. However, the antigen-specific immune response was associated with the development of anti-SUMO antibodies 6.

In current article we describe the next-generation platform where the adaptor protein SUMO was replaced with the artificial peptide pepA1, which significantly reduced the self-immunogenicity of the platform. We studied the physico-chemical properties of the novel pepA1-based platform using several target antigens and evaluated its immunogenicity in C56BL/6 mice.

## Materials & methods

### Materials

Restriction endonucleases (FastDigest), Phusion DNA polymerase (#F-530S), T4 DNA-ligase (#EL0016) and pre-stained protein markers (#26616) were purchased from Thermo Fisher Scientific, (MA, USA). All plasmids for target gene expression were constructed based on the pET28b+ vector (Novagen, WI, USA). DNA oligonucleotides were synthesized by Evrogen (Moscow, Russia). The *E. coli* TOP10 strain (Invitrogen, MA, USA) was used for plasmid propagation, and the *E. coli* strain BL21(DE3) (Novagen, WI, USA) was used for recombinant protein production. Kanamycin sulfate (#0408), LB medium (#J106), peptone 140 (#J849), and BSA (#0332) were purchased from VWR Life Science AMRESCO (PA, USA). Yeast extract (#0207) was acquired from Biospringer (Maisons-Alfort, France). Inorganic substances, EDTA, Tris, urea, Tween-20 and DTT, citric acid were purchased from Helicon (Moscow, Russia) unless specified otherwise. PMSF (BioChemica) and Lactose 1-hydrate were both purchased from Panreac Applichem (Darmstadt, Germany). Glycerol was purchased from Panreac Qu´ımica SLU, Castellar del Vall'es, Spain. NiNTA resin (#R90115) was acquired from Invitrogen. To purify the SUMO-based protein fusions (SF) proteins, we utilized the NGC chromatography system (Bio-Rad, CA, USA) with an installed HiTrap Phenyl HP column (#17-5195-01) GE Healthcare, IL, USA. Protein samples were concentrated by ultra-filtration using NMWL 5 kDa membranes (#PLCC02510) and sterilized by filtration using a Millex-GP syringe filter unit with a pore size of 0.22 μm from Millipore (Merck KGaA, Darmstadt, Germany). Complete Freund's adjuvant was kindly provided by FSBSI “Chumakov FSC R&D IBP RAS” (Moscow, Russia). Incomplete Freund's adjuvant was purchased from PanEco (Moscow, Russia). The HCT116 colon carcinoma and MCF-7 breast carcinoma cell lines were purchased from American Type Cancer Collection (Manassas, VA). Non-malignant hFB-hTERT6 skin fibroblasts were obtained via lentiviral transduction of the full-length TERT gene under a cytomegalovirus promoter (generated at Engelhardt Institute of Molecular Biology (Moscow, Russia) by E. Dashinimaev). Cell strainers 70 μM (#15-1070) and MTT reagent (3-[4,5-dimethylthiazol-2-yl]-2,5-diphenyltetrazolium bromide) (#D298931.0001) were acquired from Dia-m (Moscow, Russia). For mammalian cell culture, RPMI-1640 (#C330), Dulbecco modified Eagle's medium (#C420), fetal bovine serum (#К052m/SV30160.03), L-glutamine (#Ф032), penicillin-streptomycin (#А073), gentamycin (#A011), concanavalin A (#M011) and 96-well plates (#30096) were purchased from PanEco (Moscow, Russia). For RNA extraction, cDNA synthesis and RT-PCR, ExtractRNA reagent (#BC032), MMLV Reverse Transcriptase, 5X First strand buffer, DTT (#SK022S), Oligo(dT)_15_ primer (#SB001), dNTP mix (#PB006S), deionized water, nuclease-free (#PB207S), 5X qPCRmix-HS SYBR (#PK147L) were acquired from Evrogen (Moscow, Russia). Glycogen, RNA grade (#R0551), sodium acetate (3 M), pH 5.5, RNase-free (#AM9740), DNase I, RNase-free (1 U/μL), 10X Reaction Buffer (with MgCl2), 50 mM EDTA (#EN0521) were purchased from Thermo Fisher Scientific (MA, USA). 96-well PCR plates (#PCR-P-96-01W) were acquired from Dia-m (Moscow, Russia). Plate sealing films were purchased from PanEco (Moscow, Russia). For ELISA, 96-well high-binding plastic plates (#655061) were used (Greiner, Kremsmünster, Austria). P-GAM Iss (C = 1 mg/ml) HRP-conjugated anti-mouse goat antibodies were purchased from Imtek (Moscow, Russia). O-phenylenediamine was obtained from Sigma-Aldrich, (MO, USA).

## Methods

### Plasmid construction and protein expression

Standard methods were used to construct recombinant DNAs. The plasmids were constructed based on the pET28b+ vector using primers listed in Table 1, unless otherwise specified. The *E. coli* TOP10 strain was used for plasmid amplification. Transformants were grown at 37 °C in LB medium supplemented with kanamycin (30 μg/ml) on a rotary shaker at 250 rpm.

Some plasmids used in this work were constructed earlier, including the following:

Plasmid pET28s 6 is a derivative of the pET28b+ (Novagen) containing a unique *Sal*I site replacing the unique *Bgl*II site in pET28b+.

Plasmid pET28del-PhtD19 6 contains the unique *Bgl*II/*Xho*I DNA fragment encoding the PhtD19 peptide, derived from the gene of the pneumococcal histidine triad protein D (a.a. 200-219) 15 that is of interest as a vaccine component 6.

Plasmid pET28s-His_10_-SUMO(gg)-PhtD19 6 expressing the substrate protein His_10_-SUMO-PhtD19 was used in ELISA for specific anti-PhtD19 antibody analysis.

Plasmid pET28s-His_10_-SUMO-His_6_ 6 expresses the *S. cerevisiae SMT3* gene encoding the SUMO protein. SUMO contains the native Gly-Gly cleavage site and His_6_ tag at the C-terminus. A unique *Bam*HI site separates the sequences encoding SUMO and His_6_ in these plasmids.

Plasmid pET28s-L_6_KD-eGFP 11 contains an *Nco*I/*Xho*I DNA fragment encoding L_6_KD-eGFP protein and a unique *Bam*HI site that separates the sequences encoding L_6_KD and eGFP in this plasmid.

In addition, we used plasmid p71-66 designed in our laboratory that is a derivative of pET28b+ containing a unique site *Bam*HI between the *Nco*I and *Xho*I sites just downstream the *Nco*I (sequence ccatgggatcc…ctcgag, sites *Bam*HI and *Xho*I are underlined).

The rest of the plasmids used in this study were as follows:

An auxiliary plasmid, pET28del-Glp1G, was constructed by replacing the unique *Bgl*II/*Xho*I DNA fragment in pET28b+ with a *Bgl*II/*Xho*I fragment that was PCR-amplified from pET28-Glp20 16, 17 using primers N1727 and N619*. As a result, the unique *Bgl*II/*Bam*HI DNA fragment encoding the Glp1G peptide was inserted in the plasmid pET28del-Glp1G.

The DNA sequence encoding the pepA1 peptide was cloned in pET28b+ as a *Bgl*II/*Xho*I DNA fragment that was PCR-amplified using a mix of primers N1755, N1756, and N1757. The resulting plasmid was named pET28del-pepA1.

The DNA sequence encoding the OVA24 peptide was cloned in pET28b+ as a part of *Mlu*I/*Xho*I DNA fragment that was obtained by two-step PCR using pET28b+ as a matrix. The first step used primers N1734 and N1972, and the second step used primers N1734 and N1971. The resulting plasmid was named pET28del-OVA24. A unique *Bgl*II site separates the vector sequence and the sequence encoding OVA24 in these plasmids.

An auxiliary plasmid pET28s-gs5 was constructed by replacing the unique *Nco*I/*Xho*I fragment in pET28s with a *Nco*I/*Xho*I fragment that was PCR-amplified from pET28s using primers N1734 and gs5rc. The insertion contains the unique *Bgl*II/*Bam*HI DNA fragment encoding Gly_4_Ser linker (gs5 linker).

An auxiliary plasmid pET28del-SUMO(gg)-His_6_ was constructed by replacing the *Bgl*II/*Xho*I fragment in pET28b+ with an *Bgl*II/*Xho*I fragment that was PCR-amplified from p101-18 using primer N450 and a standard pUC18/19 reverse primer. As a result, the *S. cerevisiae SMT3* gene encoding the SUMO protein was inserted in pET28del-SUMO(gg)-His_6_; SUMO contains the native Gly-Gly cleavage site and His_6_ tag at the C-terminus. A unique *Bam*HI site separates the sequences encoding SUMO and His_6_ in this plasmid.

The auxiliary plasmids pET28-MGS-Glp1G and pET28-MGS-pepA1 were constructed by replacing the unique *Bam*HI/*Xho*I DNA fragment in p71-66 with *Bgl*II/*Xho*I fragments from plasmids pET28del-Glp1G and pET28del-pepA1, respectively. A unique *Bam*HI site separates the sequences encoding peptides Glp1G/pepA1 and site *Xho*I in these plasmids.

Plasmid pET28s-His_10_-SUMO-OVA24 was constructed for the synthesis of the substrate protein His_10_-SUMO-OVA24 used in ELISA for specific anti-OVA24 antibody analysis. For this purpose, the *Bam*HI/*Xho*I DNA fragment in plasmid pET28s-His_10_-SUMO-His_6_ was replaced with the *Bgl*II/*Xho*I fragment from plasmid pET28del-OVA24.

Plasmids pET28-Glp1G-SUMO-His_6_ and pET28-pepA1-SUMO-His_6_ were constructed for synthesis of substrate proteins Glp1G-SUMO-His_6_ and pepA1-SUMO-His_6_ used in ELISA for specific anti-Glp1 and anti-pepA1 antibody analysis, respectively. For this purpose, the *Bam*HI/*Xho*I DNA fragments in plasmids pET28-MGS-Glp1G and pET28-MGS-pepA1 were replaced with the *Bgl*II/*Xho*I fragment from plasmid pET28del-SUMO(gg)-His_6_.

Plasmids pET28s-L_6_KD-Glp1G and pET28s-L_6_KD-pepA1 expressing the platform proteins L_6_KD-Glp1G and L_6_KD-pepA1 were constructed by replacing the unique *Bam*HI/*Xho*I DNA fragment in pET28s-L_6_KD-eGFP with *Bgl*II/*Xho*I fragments from plasmids pET28del-Glp1G and pET28del-pepA1, respectively. A unique *Bam*HI site separates the sequences encoding peptides Glp1G/pepA1 and site *Xho*I in these plasmids.

An auxiliary plasmid pET28s-L_6_KD-pepA1-gs5 was constructed by replacing the unique *Bam*HI/*Xho*I DNA fragment in pET28s-L_6_KD-pepA1 with *Bgl*II/*Xho*I fragment from plasmid pET28s-gs5. A unique *Bam*HI site separates the sequences encoding peptide gs5 and site *Xho*I in these plasmids.

Plasmids pET28s-L_6_KD-pepA1-OVA24 and pET28s-L_6_KD-pepA1-gs5-PhtD19 were constructed for the synthesis of target proteins pET28s-L_6_KD-pepA1-OVA24 and pET28s-L_6_KD-pepA1-gs5-PhtD19, respectively. For this purpose, the unique *Bam*HI/*Xho*I DNA fragments in plasmids pET28s-L_6_KD-pepA1 and pET28s-L_6_KD-pepA1-gs5 were replaced with *Bgl*II/*Xho*I fragments from plasmids pET28del-OVA24 and pET28del-PhtD19, respectively.

*E. coli* strain BL21(DE3) was used for protein biosynthesis. Transformed cells were grown in Terrific Broth (TRB) medium (24 g/L yeast extract, 12 g/L soy peptone, 2 mM magnesium sulfate, 100 mM phosphate buffer at pH 7.0, 5 g/L glycerol, 2 g/L lactose, 90 mg/L kanamycin) to stationary phase, as described previously 18, 19. Lactose was used as the inducer. For protein biosynthesis, cells were grown at 37 °C with a typical growth time of 16-18 h.

### Purification of His-tagged proteins using metal-affinity chromatography

His-tagged proteins were purified as described previously 6.

### pepA1-based protein fusions purification

For purification of L_6_KD-pepA1-gs5-PhtD19 and L_6_KD-pepA1-OVA24 proteins, a special protocol was developed. In this protocol, 4 g of wet cells were harvested from 200 ml of cell culture after overnight induction. The harvested cells were sonicated in 20 mM Tris-HCl buffer (pH 8.5) containing 5 mM EDTA (buffer A) and 1 mM PMSF. Inclusion bodies (IBs) were separated by centrifugation, washed twice with 1% Triton X-100 in PBS with 5 mM EDTA, and dissolved in 20 ml of buffer A containing 4 M urea and 50% ethanol or 8 M urea for L_6_KD-pepA1-gs5-PhtD19 or L_6_KD-pepA1-OVA24 proteins, respectively (unless specified otherwise). The obtained protein solution was purified by anion exchange chromatography on a HiTrap Q column under denaturing conditions. The target denatured protein did not bind to the sorbent and left the column in the breakthrough fraction, while some of the protein impurities were eluted with a buffer additionally containing 1 M sodium chloride. For renaturation, the resulting protein solution was diluted 10-fold with buffer A and incubated at 4 °C overnight. After renaturation, the fraction was centrifuged at 16,000 ×g for 15 min, washed from residual urea, and concentrated using an Amicon Ultra Centrifugal Filter Unit through a membrane with a molecular weight cutoff of 10 kDa. After that the protein was rechromatographed on a HiTrap Q column equilibrated with buffer A. The target renatured protein was bound to the sorbent and eluted from it with buffer A containing 0.5 M sodium chloride. The obtained protein samples were sterilized by filtration using 0.22 μm filters. For further analysis of physical and immunological properties, the protein concentrations in all samples were evenly adjusted to 0.3 mg/ml. The ready samples allowed storage at +4 °C for at least 2-3 months without deterioration, as well as lyophilization. Immediately before use, the proteins were transferred to PBS via ultrafiltration.

### SDS-PAGE

Proteins were separated using 15% PAGE under reducing and denaturing conditions (SDS-PAGE) as described earlier 17, 20. Prestained protein markers were used.

### Transmission electron microscopy

Transmission electron microscopy of negatively stained samples was carried out according to the method 6.

### Dynamic light scattering

Particle size (hydrodynamic radius/diameter) was determined with a Malvern Zetasizer Nano ZS analyzer (He-Ne laser with a wavelength of 632.8 nm, 173° measurement angle), using software supplied with the instrument. Plastic cuvettes with an optical path of 10 mm were used.

### Mass-spectrometric analysis

Mass-spectrometric analysis of protein samples was performed on an UltrafleXtreme MALDI time-of-flight mass spectrometer (Bruker Daltonics, MA, USA) equipped with a UV laser (Nd). The mass spectra were obtained in the linear positive-ion mode with a reflectron. The average m/z accuracy was 5 Da. The Vector NTI software package (Thermo Fisher Scientific) was used to calculate the protein masses.

### Small-angle X-ray scattering (SAXS) and data processing

Small-angle X-ray scattering experiments were carried out at the BioMUR beamline 21, 22 of the Kurchatov synchrotron radiation source (National Research Center Kurchatov Institute, Moscow, Russia) in transmission geometry. Sample solutions were placed into quartz capillaries with 2 mm diameter and 0.01 mm wall thickness. Total photon flux at the sample was 1.86x1010 ph/s. A two-dimensional DECTRIS Pilatus3 1M pixel-array system with an active surface area of 168.7 × 179.4 mm, resolution 981 × 1043 pixels and 0.172 mm pixel size was used as a detector for recording SAXS patterns. The detector was placed at a distance of approximately 700 mm from the sample. Scattering intensity I(s) was measured in the range of scattering vectors 0.14 < q < 6 nm^-1^, where q = (4πsinθ)/λ, 2θ is the scattering angle and λ = 0.1445 nm is the wavelength at the BioMUR beamline. Experimental scattering patterns were recorded with exposure time 500 s each. Sample-to-detector distance was calibrated with the Fit2D software 23 using silver behenate as a standard (Sigma-Aldrich, Germany). Primary data processing including subtraction of the signal from the buffer solution and determination of SAXS structural parameters was carried out using PRIMUS software 24. Further data processing was carried using the special software kit ATSAS 25. GNOM program was used for determination of the maximum size of scattering particles in solution and calculation of the distance distribution functions p(r) 26.

### Peptide synthesis

The peptides OVA_265-280_ (OVA16) and OVA_257-280_ (OVA24) were synthesized by the solid phase method using N-dimethylformamide as solvent (DMF, Scharlau, Germany), the resin of Rink-amide ChemMatrix, and Fmoc-protected amino acids (Val, Lys(Boc), ILE, Arg(Pbf), Glu(t-Bu), Met, Asn(Trt), Ser(t-Bu), Thr(t-Bu), Trp(Boc), Leu, Phe (IRIS, Germany). The O-(benzotriazol-1-yl)-N,N,N′,N′-tetramethyluronium tetrafluoroborate (TBTU) was used to form peptide bonds (Acros Organics, Belgium).

The resulting peptides were cleaved from the resin with trifluoroacetic acid (TFA, Carl Roth, Germany) in the presence of scavengers 1,2-ethanditiol and thioanisole (Sigma, USA). The raw peptide product was precipitated with dry methyl tert-butyl ether and extracted with aqueous acetic acid. Further, the peptides were purified by an ion-pairing HPLC (gradient of acetonitrile - 0.1% aqueous TFA) using a Prominence preparative chromatograph LC-20 (Shimadzu, Japan) equipped with a reversed-phase column Kromasil EternityXT-10-C18, 30x250 mm (Kromasil, Germany) and UV-detector. Molecular masses of peptides obtained were confirmed by mass spectrometry with the MALDI Bruker Microflex LT spectrometer (α-Cyano-4-hydroxycinnamic acid as a matrix). The resulting peptides were also analyzed for homogeneity by zone capillary electrophoresis with a Kapel-105M device (Lumex, Russia) with photometric detection at 226 nm using an unfilled quartz capillary and a solution of 0.1 M phosphoric acid and 0.05 M Tris in deionized water as a background electrolyte. The purity of resulting peptides was found to be 93 and 94% for OVA16 and OVA24, respectively.

### Cytotoxicity assay

SAPN-PhtD19 or SAPN-OVA24 were reconstituted in saline as stock suspensions immediately before the experiments. HCT116 colon carcinoma, MCF-7 breast carcinoma cells and non-malignant hFB-hTERT6 skin fibroblasts (5x10^3^ in 190 µl of Dulbecco modified Eagle's medium supplemented with 10% FBS, 2 mM L-glutamine, 100 U/ml penicillin, and 100 µg/ml streptomycin) were plated into a 96-well plate overnight and then treated with each nanoparticle preparation (20-200 µg/ml final concentrations, each in triplicate) for 72 h at 37 °C, 5% CO_2_. After the completion of exposure, 50 µg of 3-(4,5-dimethylthiazol-2-yl)-2,5-diphenyltetrazolium bromide was added into each well for an additional 2 h. Formazan was dissolved indimethyl sulfoxide, and the absorbance at 540 nm was measured on a Tecan Spark spectrophotometer (Tecan, Switzerland). Cell viability at a given concentration was calculated as the percentage of absorbance in wells with nanoparticle-treated cells to that of untreated cells (100%).

### Animals

The C57BL/6 male mice were provided by the breeding facility “Stolbovaya” (Moscow Oblast, Russia) and housed at 20-23 °C on a 12 h light/dark cycle with water/food supply ad libitum. Animals were used for the experiments at the age of 12 weeks. Animal manipulations were carried out in accordance with recommendations in the Guide for the Care and Use of Laboratory Animals (NRC 2011), the European Convention for the Protection of Vertebrate Animals Used for Experimental and Other Scientific Purposes, Council of Europe (ETS 123), and “The Guidelines for Manipulations with Experimental Animals” (the decree of the Presidium of the Russian Academy of Sciences of April 2, 1980, no. 12000-496). Permission to work with mice was granted by the Bioethics Committee of the NRC “Kurchatov Institute”.

### Immunization

All administered samples were >95% pure by protein composition and sterilized using 0.22 μm filters. On Days 0 and 21, each mouse in each experimental group was injected intraperitoneally with 50 μg of SAPN-PhtD19 or SAPN-OVA24 (each in 200 μl PBS) or the equivalent dose of the OVA24 peptide in 100 μl PBS mixed with an equal volume of complete (for the first immunization) or incomplete Freund's adjuvant (for the second immunization). In the control group mice were injected with PBS. On Day 28, mice were euthanized, blood was gained by cardiac puncture and serum was obtained. Spleens were collected for subsequent immune cell isolation.

### Evaluation of antibody response

To evaluate the antibody titer, direct ELISA was performed. Typically, 96-well plates were used. The wells were coated overnight at 4 °C with 1 μg of the purified fusion proteins His_10_-SUMO-PhtD19, pepA1-SUMO-His_6_, His_10_-SUMO-OVA24 or Glp1G-SUMO-His_6_. PBS (pH 7.5) containing 0.05% Triton X-100 and 0.1% BSA (buffer PBS-TB) was used for blocking. The serum samples were diluted with PBS-TB and applied to plates at 37 °C for 1 h. HRP-conjugated anti-mouse goat antibodies, diluted 1: 5000 with PBS-TB, were applied to each well at 37 °C for 1 h. The chromogenic reaction was initiated by adding 100 μl of 0.4 mg/ml O-phenylenediamine, 0.03% H_2_O_2_, phosphate citrate buffer (pH 4.5) for 10 min at room temperature and stopped by adding an equal volume of 10% H_2_SO_4_. The absorbance at 492 nm was measured using Modulus™ II Microplate Multimode Reader. Sera with OD values greater than 2.1× the mean OD value of the negative control was defined as antibody-positive (seropositive). Each antibody titer was established as the reciprocal of the sample dilution, giving a significant value. Geometric mean titers of antibodies (GMT) were calculated. The seroconversion rate was calculated as the percentage of mice with seropositive sera against specific antigen targets.

### Spleen cell isolation

Spleens were placed on ice and mechanically mashed through a 70-μm cell strainer into a 60 mm Petri dishes with ice-cold PBS/2%FBS. Cell suspension was transferred to a 15 ml centrifuge tube and centrifuged at 300 g for 7 minutes at 4 °C. Supernatant was discarded and the cell pellet was resuspended in 1 ml of 37 °C ACK lysis buffer (15 mМ NH_4_Cl, 1 mM KHCO_3_, 0.01 mM EDTA, pH 7.3). After lysing at room temperature for 3 minutes, 14 mL of ice-cold PBS/2%FBS were added and samples were centrifuged as described previously. After removal of supernatant, cells were resuspended in 1 ml of complete RPMI 1640 medium (RPMI 1640 medium supplemented with 10% FBS, 2 mM L-glutamine and 50 μg/ml gentamycin) and counted using a haemocytometer.

### Cytokine expression assay

Splenocyte concentration was adjusted to 5×10^6^ cell/ml, after which 1 ml of cell suspension was transferred into a 24-well plate and treated with either vehicle, concanavalin A (ConA, 5 μg/ml) or OVA24 peptide (10 μg/ml). Following a 6-hour incubation at 37 °C with 5% CO_2_ samples were transferred to 1.5 ml centrifuge tubes and centrifuged at 300 g for 7 minutes at room temperature. After removal of supernatant, cells were resuspended in 500 μl of ExtractRNA reagent and stored at -80 °C. RNA was isolated using phenol-chloroform extraction. Briefly, frozen-thawed samples were incubated for 5 minutes at room temperature, after which 100 μl of chloroform was added. Following a 3-minute incubation at room temperature, the tubes were centrifuged at 12,000 g for 15 minutes at 4 °C. The upper aqueous phase containing the RNA was transferred to a new tube. To precipitate the RNA, 1 μg of RNAse-free glycogen, 300 μl of 3 M sodium acetate, and 250 μl of isopropanol were added. Samples were incubated for 10 minutes at 4 °C and centrifuged at 12,000 g for 10 minutes at 4 °C. After removal of supernatant, the pellet was resuspended in 500 μl of 75% ethanol and centrifuged at 7,500 g for 5 minutes at 4 °C. The supernatant was discarded, and the RNA pellet was air-dried for 15-20 minutes and resuspended in 15 μl of nuclease-free water. Purity and concentration of RNA were measured using a NanoDrop ND-1000 instrument. RNA concentration was adjusted to 250 μg/ml. cDNA synthesis was performed using a SimpliAmp™ Thermal Cycler. Briefly, 8.75 μl of the RNA sample were transferred to the 0.2 ml PCR tube, and 0.25 μl of DNAse I and 1 μl of 10x Reaction Buffer (with MgCl_2_) were added. Samples were incubated for 30 minutes at 37 °C to remove the genomic DNA, then 1 μl of 50 mM EDTA was added, and the reaction was terminated by heating at 65 °C for 10 minutes. 1 μl of 100 μM Oligo(dT)_15_ primer was transferred to the tube and samples were incubated for 5 minutes at 65 °C. Then 4 μl of 5X First strand buffer, 2 μl of dNTP mix, 2 μl of DTT, and 1 μl of MMLV Reverse Transcriptase were added, and samples were incubated for 60 minutes at 42 °C, after which the reaction was terminated by heating at 70 °C for 5 minutes. Quantification of *Ifng*, *Il2* and *Il4* and the internal reference gene *Actb* was performed using a RT-PCR (CFX96 Touch Real-Time PCR Detection System, Bio-Rad). The cDNA samples were diluted 2.5 times and transferred to a 96-well PCR plate. The PCR reaction mixture contained 4 μl of cDNA, 4 μl of 5X qPCRmix-HS SYBR, 1 μl of mixed forward and reverse primers (10 μM each), and 11 μl of nuclease-free water. The primers sequences used are listed in Table 2. Cycling conditions were 50 °C for 2 minutes, 95 °C for 10 minutes, followed by 45 cycles at 95 °C for 15 seconds and 60 °C for 1 minute. The relative fold gene expression was calculated using the 2^-∆∆Ct^ method 27.

### MTT-assay

Splenocyte concentration was adjusted to 2.5×10^6^ cell/ml, after which 200 μl of cell suspension was transferred into a 96-well plate and treated with either vehicle, ConA (5 μg/ml) or OVA24 peptide (10 μg/ml). Following 45-hour incubation at 37 °C with 5% CO2, 50 μl of 1 μg/ml MTT reagent was added, and samples were incubated for another 2.5 hours. Samples were centrifuged at 400 g for 10 minutes at room temperature. After removal of supernatant, 100 μl of DMSO was added and samples were incubated for 10 minutes at room temperature. The absorbance at 570 nm was measured using iMark™ Microplate Absorbance Reader, Bio-Rad. Cell proliferation was calculated by dividing absorbance in wells with treated cells to that of untreated cells.

### Statistical analysis

Data were analyzed using Prism software (GraphPad). The serum IgG titers were log-transformed approximate a normal distribution. Normality was assessed using the Shapiro-Wilk test. Comparisons of serum IgG titers between groups were performed using the unpaired, two-tailed t-test with unequal variances. Differences in cytokine mRNA expression and cell proliferation were evaluated using ordinary one-way ANOVA with Tukey's correction for multiple comparisons or Kruskal-Wallis test with Dunn's correction for multiple comparisons depending on whether the data followed a normal or non-normal distribution, respectively. A p-value of < 0.05 was considered statistically significant.

## Results

### Design of pepA1-based fusions

In our previous work, we showed that the L_6_KD-SUMO platform enables the synthesis of self-assembled protein nanoparticles (SAPN) characterized by approximately equal immunogenicity of the adaptor protein SUMO and target antigen PhtD19 6. In order to reduce the immune response to the platform protein, we searched for an alternative adaptor with a shorter amino acid sequence. To this end, various trial peptides from the laboratory collection were tested as a part of L_6_KD-fusions for their ability to promote the synthesis of protein constructs in *E. coli* and support the self-assembly of SAPN.

Of those, only modified glucagon-like peptide 1 Glp1 with Ala8Gly substitution 17 and human ubiquitin showed appropriate functional activity. However, given the fact, that application of these highly conserved mammalian proteins as part of SAPN can potentially trigger the autoimmune response, we aimed to obtain a highly modified derivative of Glp1(Ala8Gly). For this purpose, we designed candidate peptides pepA1, pepA2, and pepA3, which differed from the original in at least nine amino acid residues (Table 3).

When developing modified variants of the Glp-1(Ala8Gly) adapter peptide, our goal was to generate sequences with a high number of amino acid substitutions while ensuring that the mutant variants retained the key structural features of the original peptide, Glp-1(Ala8Gly). We employed different strategies for this purpose. For the design of pepA1, we utilized a straightforward approach where amino acid residues in the sequence Glp-1(Ala8Gly) were replaced by the related ones, namely KóR, EóD, SóT, QóN, FóW and additionally HóK. In contrast, the design of pepA2 and pepA3 involved using secondary structure modeling to guide substitutions. We selected mutant variants that maintained the highest structural similarity to Glp-1(Ala8Gly). The structural models were generated using specialized RaptorX web server 28.

**Figure 1:**
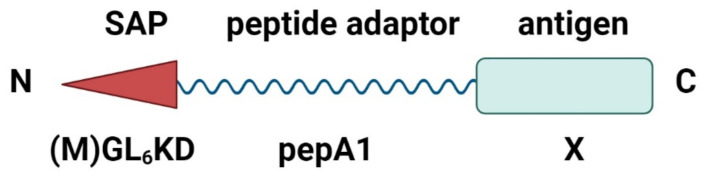
** Schematic structure of the pepA1 fusion constructs.** The fusion constructs were designed to contain the N-terminal self-assembling peptide (SAP), the adaptor peptide pepA1, responsible for the fusion construct expression, and the C-terminal antigen. Created with BioRender.com.

The experimental examination of the candidate peptides showed that pepA1 was the only one which inherited relevant functional properties of the Glp-1(Ala8Gly) and enabled effective synthesis of protein fusions (Figure S1*a*). In contrast, the other peptides did not facilitate the synthesis of protein constructs in *E. coli* (Figure S1*b*). Consequently, to minimize the risk of cross-reactivity between nanoparticles and the natural hormone Glp-1, the artificial derivative pepA1 was selected for further development of the platform (Figure 1).

### Biosynthesis and extraction of pepA1-based protein fusions

**Figure 2:**
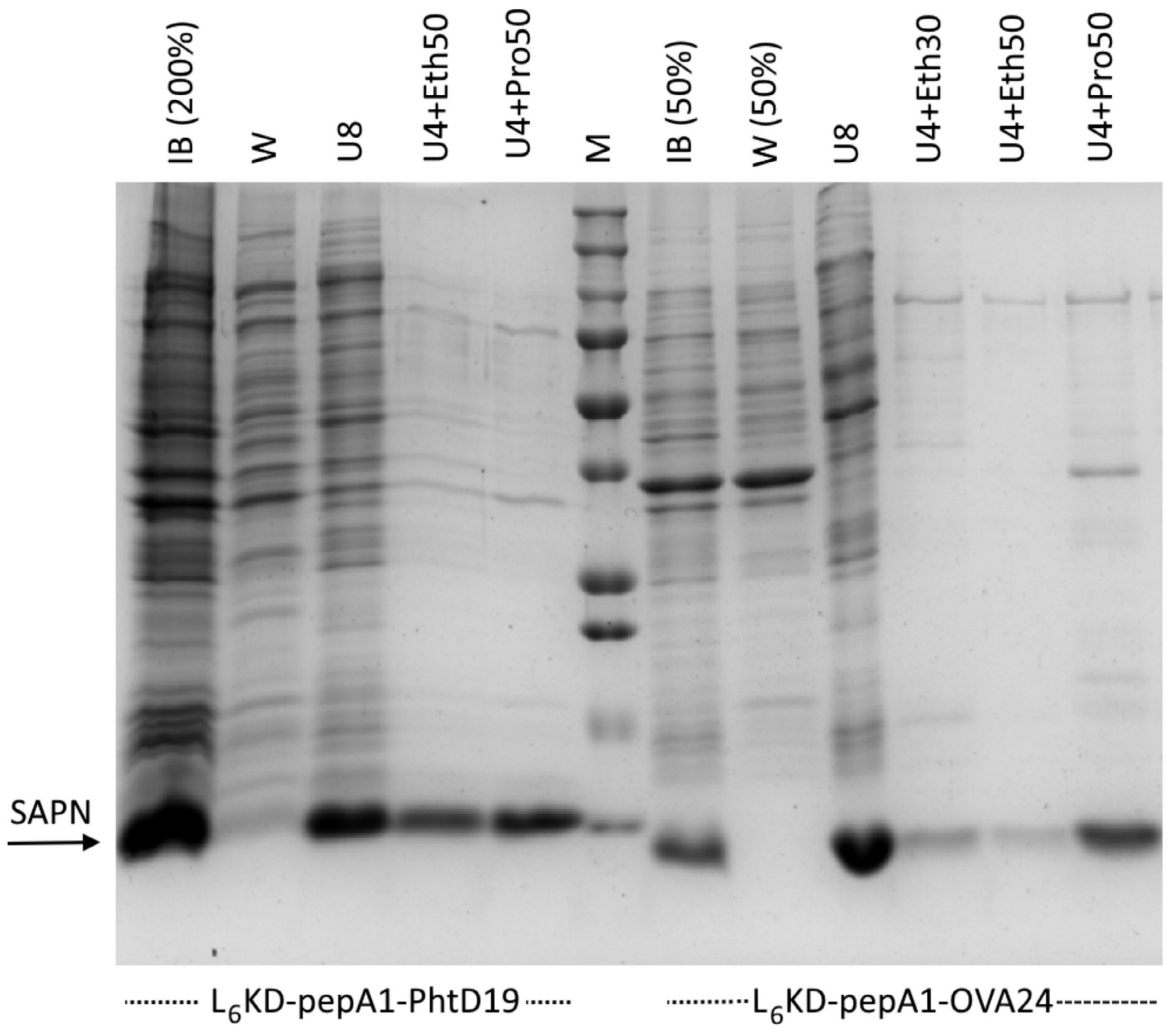
** Purification of L_6_KD-pepA1-gs5-PhtD19 and L_6_KD-pepA1-OVA24 fusion proteins synthesized in E. coli BL21(DE3) cells.** Analysis was performed via 15% SDS-PAGE. Proportional amounts of samples were loaded into the wells (deviations from the proportion are indicated in parentheses). We analyzed proteins from the inclusion bodies, insoluble fraction of cell lysate (IB), the IB wash solution (W), and the proteins from washed IB dissolved in 8 M urea (U8) or in a mixture of 4 M urea and 50% or 30% ethanol (U4+Eth50 or U4+Eth30) or in a mixture of 4 M urea and 50% isopropanol (U4+Pro50). SAPN fusion proteins are indicated by arrows. M - molecular weight markers (BioRad 161-0373, MW 250, 150, 100, 75, 50, 37, 25, 20, 15, 10 kDa).

We generated two types of pepA1-based protein fusions, carrying either PhtD19 or OVA24 peptides as the target antigens (L_6_KD-pepA1-gs5-PhtD19 and L_6_KD-pepA1-OVA24, respectively). The first, a pneumococcal histidine triad protein D fragment, was previously tested as a part of the SUMO-based SAPN 6, which allowed us to make a comparison of two platforms. The second represents the 24-amino acid fragment of the chicken ovalbumin (OVA_257-280_), widely used in immunological studies 29. Both fusion constructs were synthesized with high efficiency as inclusion bodies in *E. coli* BL21(DE3) strain (Figure 2).

For L_6_KD-pepA1-gs5-PhtD19 and L_6_KD-pepA1-gs5-OVA24 fusion protein purification, we developed a selective extraction step that showed high efficiency for some other proteins designed on the L_6_KD-pepA1 platform. Inclusion bodies (IB) isolated from *E. coli* cells and containing target proteins were subjected to extraction with two-component mixtures containing a chaotropic agent, urea (usually 4 M), and a water-soluble hydrophobic phase (alcohols, acetonitrile, acetone). The selective extraction resulted in the transfer of the target protein to the soluble phase, while most of the impurity proteins remained insoluble. Compared to the non-selective extraction of IB proteins with a solution of 8 M urea, the novel method enabled us to both maintain a high yield of the L_6_KD-pepA1-gs5-PhtD19 fusion protein, and to significantly increase the selectivity of the process (Figure 2). The method's efficiency varied depending on the protein construct, and its optimization required careful selection of the extraction mixture composition as well as the extraction conditions. In particular, the extraction of L_6_KD-pepA1-gs5-OVA24 protein with the mixture of 4 M urea and 50% ethanol led to the noticeable loss of the target protein as compared to the usage of 8 M urea (Figure 2).

The complete protocol for purification of the target proteins and preparation of corresponding samples of SAPN is described in the Materials and Methods section.

**Figure 3:**
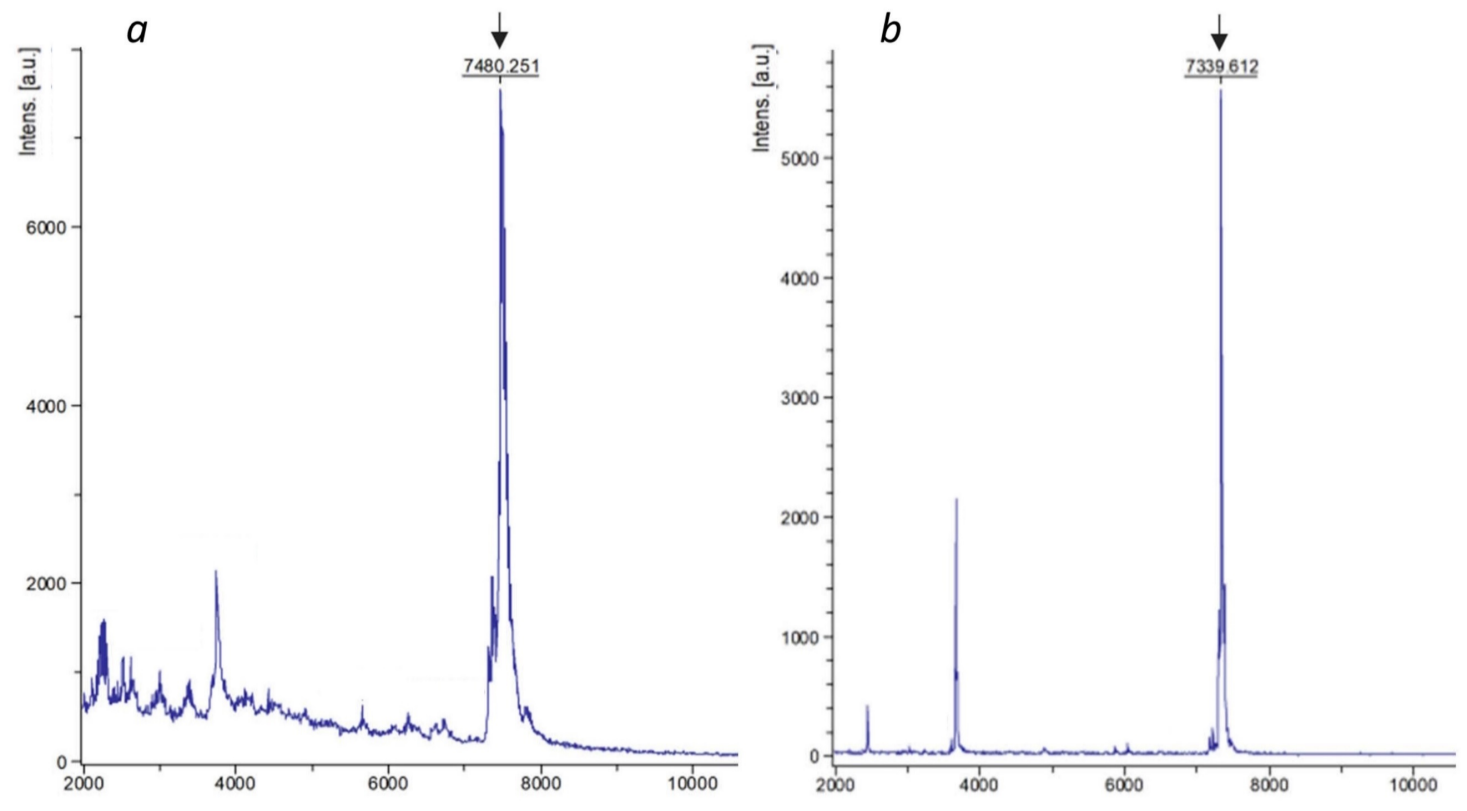
** Mass-spectrometry analysis of SAPN-proteins.** L_6_KD-pepA1-OVA24 (a) and L_6_KD-pepA1-gs5-PhtD19 (b) fusion proteins were analyzed via 15% SDS-PAGE mass-spectrometry. The peaks corresponding to the target products are pointed out by arrows.

In order to evaluate the integrity of the purified SAPN, samples were analyzed by mass spectrometry. We found out that despite the high spectrometric purity of the resulting fusion proteins, their molecular weight was 160.8±0.5 Da higher than the corresponding calculated values (Figure 3, Table 4). Taking into account the molecular weight of methionine (131.2 Da) and formylmethionine (159.2 Da), as well as the expected instrument error (2 Da), we assumed that the pepA1-based fusion proteins retain N-terminal formylmethionine, which was not observed for SUMO-based SAPN 6. We speculated that it can be explained by the relatively low length of the pepA1-based fusion proteins, which impedes efficient N-terminal processing 30.

### Basic analysis of purified SAPN

In order to determine the physicochemical properties, pepA1-based preparations of SAPN-PhtD19 and SAPN-OVA24 were analyzed via negative contrast transmission electron microscopy (TEM), dynamic light scattering (DLS), and small-angle X-ray scattering (SAXS).

**Figure 4:**
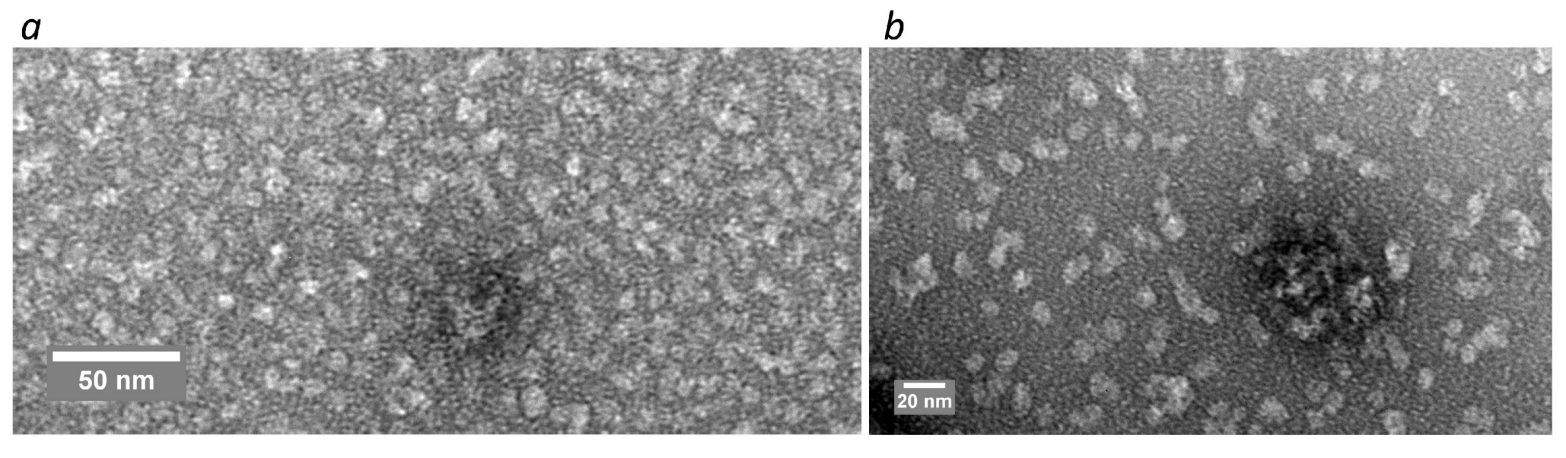
TEM analysis of SAPN-OVA24 (*a*) and SAPN-PhtD19 (*b*).

The TEM results showed that both studied SAPN samples were mainly represented by spherical particles with a diameter of less than 10 nm (Figure 4), which distinguishes them from SUMO-based SAPN tending to form nanofibers 6.

**Figure 5:**
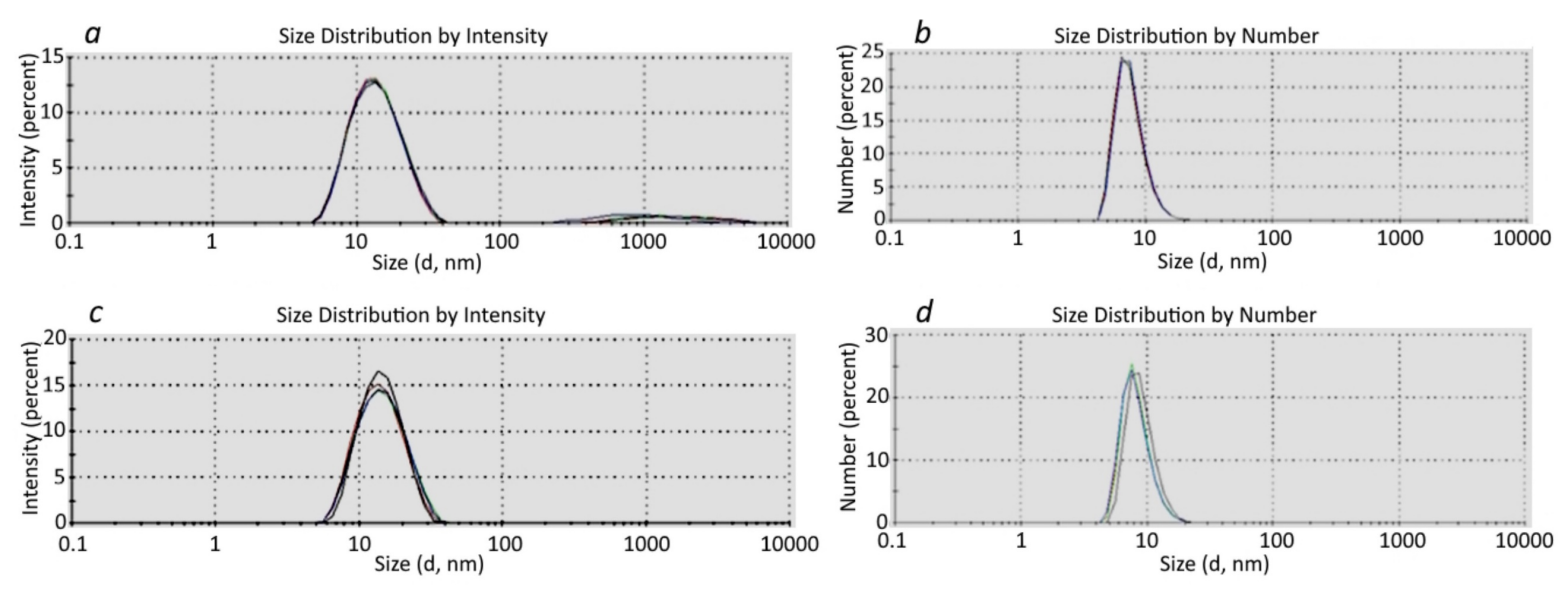
** DLS analysis of SAPN-OVA24 (a, b) and SAPN-PhtD19 (c, d).** Each graph represents the sum of 4 independent measurements.

The DLS results were consistent with the TEM data (Figure 5). In addition, the DLS analysis confirmed the purity of the SAPN samples and showed that the SAPN-PhtD19 specimen was more homogeneous compared to the SAPN-OVA24 sample, which contained some high-molecular aggregates (Figure 5).

At the same time, we believe that the DLS results should be considered with caution, since the distribution by intensity gave a somewhat higher estimation of the SAPN size compared to the distribution by number (Figure 5).

We calculated the SAPN average hydrodynamic radius and mass, which enabled us to estimate the number of monomers with a known mass that formed the corresponding nanoparticles (Table 5). In particular, we showed that SAPN-OVA24 contained approximately 40 monomeric subunits, whereas SAPN-PhtD19 comprised 20-40 monomers. The data given below refer to the SAPN fractions that formed the main peak in the particle number versus size distribution.

**Figure 6:**
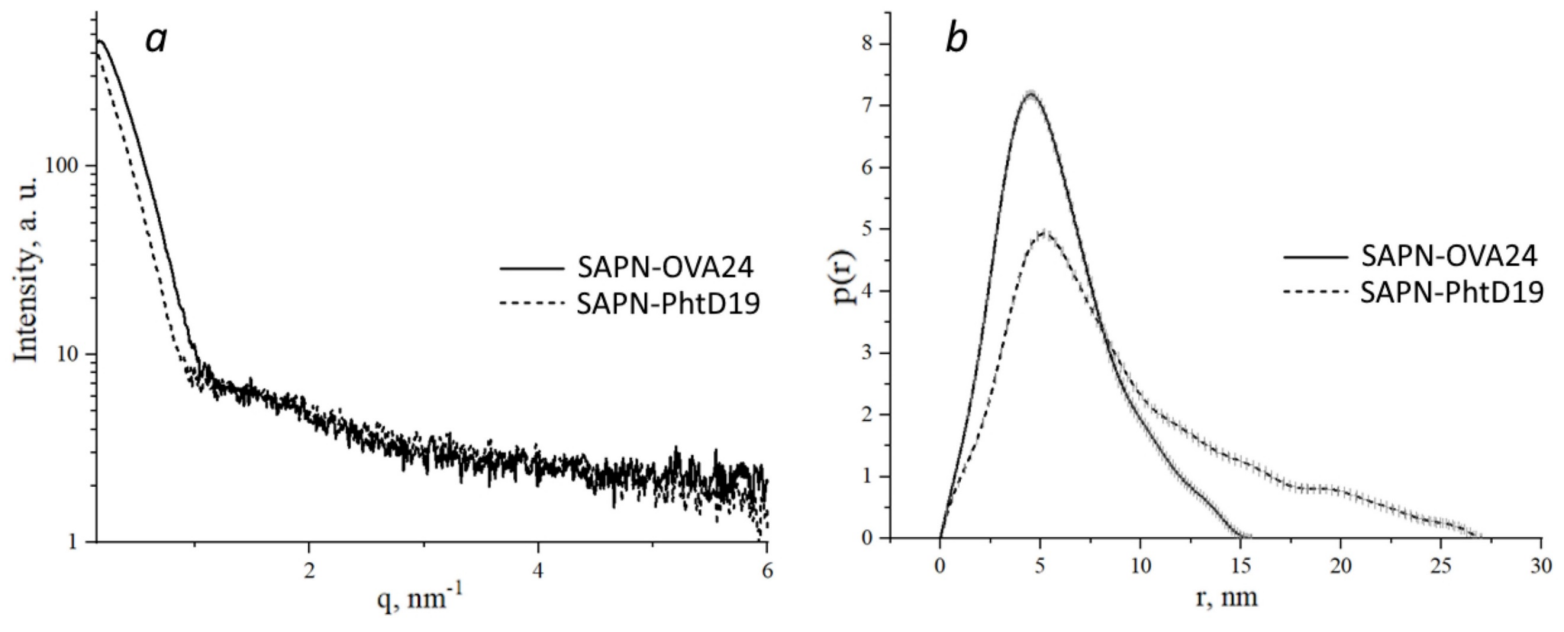
** SAXS analysis of SAPN-OVA24 and SAPN-PhtD19.** a - SAXS intensity curves. Experimental scattering curves were obtained after subtracting the signal from the buffer solution. b - Distance distribution functions obtained using the GNOM software.

We also performed small-angle X-ray scattering (SAXS) analysis of pepA1-based SAPN. Processing of the scattering curves (Figure 6a) showed that in the Guinier approximation in the region of small angles, the SAPN radius of gyration was 6.97±0.02 nm and 5.72±0.04 nm for SAPN-OVA24 and SAPN-PhtD19, respectively.

Based on the primary SAXS data, we constructed the pair distance distribution functions p(r), reflecting the shape of macromolecules (Figure 6b). We showed that SAPN-OVA24 were more compact than SAPN-PhtD19. Moreover, both specimens contained non-globular aggregates, since particles whose size was several times larger than the radius of gyration, made a significant contribution to the distribution function.

The distribution functions for SAPN-OVA24 and SAPN-PhtD19 showed maximum at points 4.5 nm and 5.16 nm and approached zero at points 15.65 nm and 27.26 nm, which corresponded to the most frequent and maximum possible structure size, respectively.

In addition to the physicochemical properties, we evaluated the SAPN nonspecific cytotoxicity, which determines the prospects for their later practical application. The cytotoxic properties of pepA1-based nanoparticles were assessed in human HCT116 colon carcinoma cell line, MCF-7 breast carcinoma cell line, and non-malignant hFB-hTERT6 skin fibroblasts by MTT-assay. No cytotoxicity was observed after 72 h incubation with 20-200 µg/ml of each nanoparticle preparation, demonstrating their safety (Figure S2).

### Induction of antibody response in mice

**Figure 7:**
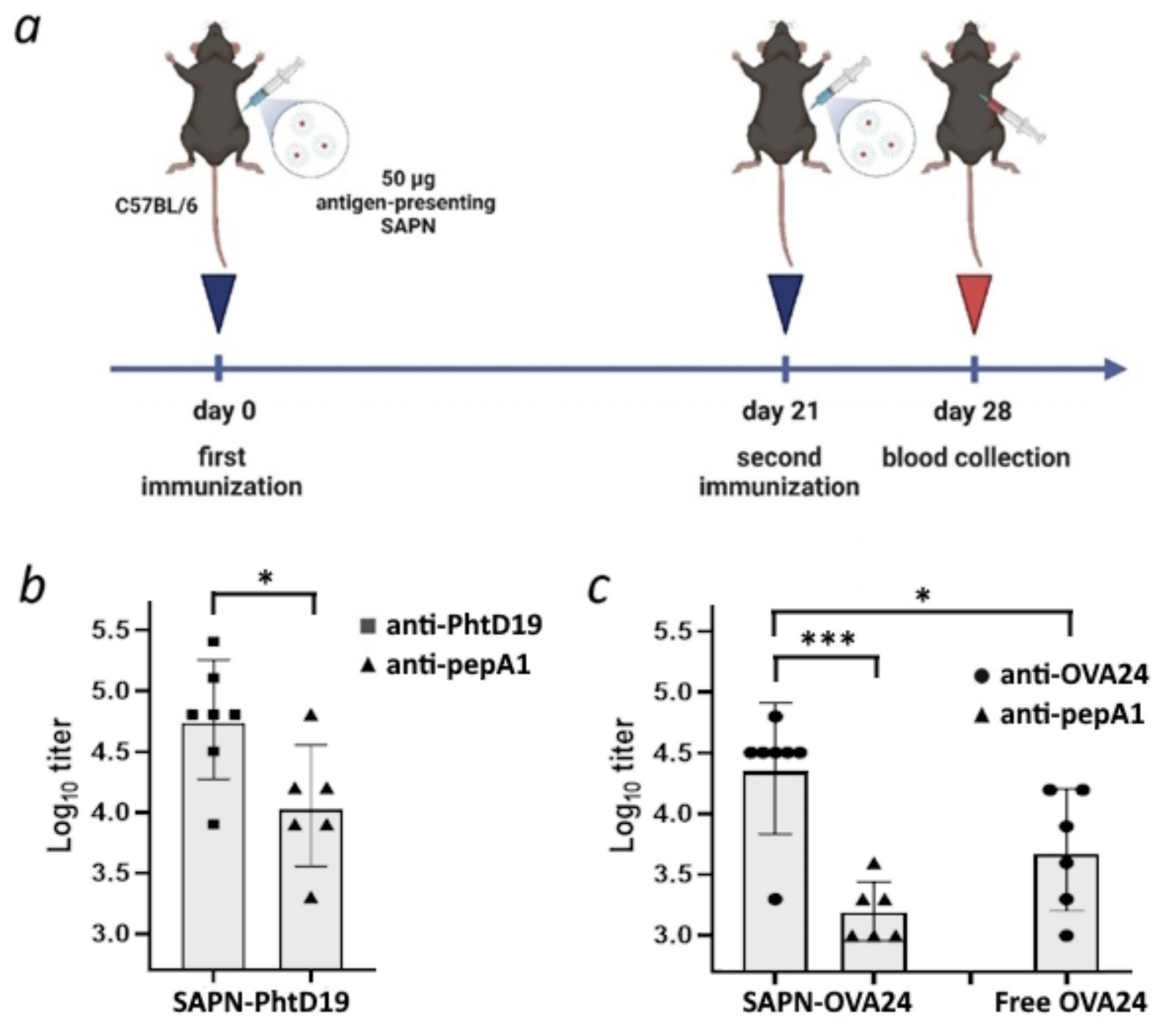
** pepA1-based SAPN induce the antigen-specific IgG response in C57BL/6 mice.** a - 12-week C57BL/6 male mice were immunized intraperitoneally on day 0 and day 21 with equal amounts of the following compounds each time: 50 μg SAPN per animal in the absence of adjuvants, the equivalent dose of the free peptide antigen mixed with an equal volume of complete (for the first immunization) or incomplete Freund's adjuvant (for the second immunization) or phosphate-buffered saline (negative control). Serum was collected on day 28 and analyzed by ELISA. Created with BioRender.com. b - Mice were immunized with SAPN-PhtD19 and serum IgG titers were tested against the His_10_-SUMO-PhtD19 and pepA1-SUMO-His_6_ coating antigens. c - Mice were immunized with SAPN-OVA24 or free OVA24 peptide, and serum IgG titers were tested against the His_10_-SUMO-OVA24 and pepA1-SUMO-His_6_ coating antigens. Data are presented as geometric mean and geometric standard deviation. *p < 0.05, ***p < 0.001.

To compare the antibody-inducing capacity of our novel pepA1-based biosynthetic platform with the previously reported SUMO-containing nanoparticles 6, we immunized C57BL/6 mice with pepA1-based SAPN-PhtD19 carrying the pneumococcal histidine triad protein D fragment (a.a. 200-219) 15 referred to as PhtD19, as described previously 6. Briefly, mice were injected intraperitoneally with adjuvant-free SAPN-PhtD19 or vehicle on days 0 and 21, and one week later serum was collected (Figure 7a). The interval of one week for serum collection after booster injection was selected as a compromise to expedite the experiments, given that precise optimization of the immunization protocol was considered impractical at the current stage of research. The titer of PhtD19- and pepA1-specific IgG was determined using ELISA.

We showed that SAPN-PhtD19 drove the PhtD19-specific IgG response with 100% seroconversion rate (Figure 7b). Moreover, the PhtD19-specific IgG response significantly exceeded (p < 0.05) the non-specific response to pepA1 (Fig. 7b). The low level of antibodies recognizing the pepA1 peptide (Figure 7b) proves the poor immunogenicity of the platform, leading us to conclude that the pepA1-based SAPN is an improvement on the previously reported SUMO-based SAPN.

To test whether the pepA1-based nanoparticles can serve as a universal platform for vaccine development, we generated SAPN carrying the ovalbumin fragment (a.a. 257-280) referred to as OVA24 29 (SAPN-OVA24) and used them for mouse immunization as described above. We found that SAPN-OVA24 induced the generation of OVA24-specific IgG in mice, while only a significantly weaker (p < 0.001) antibody response to the pepA1 peptide was detected (Figure 7c). Moreover, the adjuvant-free SAPN-OVA24 showed much higher immunogenicity against the target antigen than the equivalent dose of the free OVA24 peptide in Freund's adjuvant (p < 0.05, Figure 7c). The obtained results suggest that pepA1-based SAPN represent a promising platform for future vaccine development.

### Analysis of T-cell response

The induction of T cell-mediated immunity is crucial for the development of a robust and specific antibody response. Therefore, an efficient vaccine should boost both cellular and humoral immunity 31. To evaluate whether our SAPN induced the T cell response, we isolated splenocytes from C57BL/6 mice immunized with SAPN-OVA24 and from control animals, incubated cells with either the free OVA24 peptide or a vehicle control, and assessed the expression of cytokines related to the Th1 and Th2 immune responses 32. We did not observe upregulation of cytokine mRNA expression in splenocytes isolated from the immunized animals and activated with the OVA24 peptide as compared to control mice (Figure S3). Furtermore, we did not detect the OVA24-mediated splenocyte proliferation using the MTT assay (Figure S4). This data may suggest that our biosynthetic platform fails to elicit a strong antigen-specific T cell response in mice following the two-time immunization.

## Discussion

Previously, we reported about the proof-of-concept development of a new L_6_KD-SUMO platform for the presentation of antigens on the surface of self-assembled protein nanoparticles (SAPN) 6. We showed that two-shot immunization with nanoparticles carrying the pneumococcal protein fragment PhtD19 induced a robust antigen-specific antibody response in C57BL/6 mice. This result allows us to consider the platform as a promising tool for vaccine engineering 6. However, the relatively high immunogenicity of SUMO itself 6 could impede the further application of the platform for vaccine development. For that reason, we aimed to modify the platform by substituting the SUMO protein with a less immunogenic adaptor.

In the current work, we designed the second generation of SAPN, where the SUMO protein was replaced with the artificial peptide pepA1, a derivative of the human hormone Glp1. The fusion proteins L_6_KD-pepA1-gs5-PhtD19 and L_6_KD-pepA1-OVA24, developed on the basis of the updated L_6_KD-pepA1 platform and containing the target peptides PhtD19 and OVA24, demonstrated efficient production in *E. coli*, comparable to that of the previous generation L_6_KD-SUMO platform.

Utilizing SAPN for antigen exposure, we aimed to evolve the biosynthetic platform towards improving the ratio of its size to the size of the target peptide. In this regard, we assume that (i) an excessive reduction in the size of the platform could negatively affect biosynthesis efficiency due to the susceptibility of short peptides to proteolysis 33; (ii) intracellular degradation of the SUMO-based nanoparticles was impeded due to the presence of the full-length protein in their composition. Our results supported this hypothesis. The substitution of SUMO with a case adaptor peptide almost always reduced the SAPN synthesis to zero. Among the many short peptides tested, only the modified human hormone Glp1 and its derivative pepA1 provided efficient synthesis of the peptide constructs in *E. coli* (Figure S1). We speculate that this phenomenon can be at least partly explained by the ability of Glp1 to interact with each other and form low-molecular-weight oligomers 34. In particular, the intermolecular interactions of Glp1 (or pepA1) could accelerate the self-assembly of synthetized target proteins, thus preventing their nonspecific degradation in bacterial cells. Previously, such *in vivo* self-assembled structures were found in *E. coli* synthesizing L_6_KD-SUMO fusions 11. Moreover, it was the Glp1/pepA1-mediated additional interaction between subunits that could ensure SAPN resistance to ultrasonic waves, which was not the case with SUMO-based nanoparticles 11. However, the exact mechanism of target protein synthesis in bacterial cells and the functional role of the Glp1/pepA1 in this process requires further investigation.

One additional aspect related to the miniature pepA1 platform application for the synthesis of target proteins in *E. coli* concerns the N-terminal formylmethionine. This modification was unexpectedly found in both target proteins, L_6_KD-pepA1-gs5-PhtD19 and L_6_KD-pepA1-OVA24. Moreover, the mass-spectrometric analysis of the corresponding SAPN preparations did not reveal any completely processed target proteins at all. We are currently unable to offer any mechanism to explain this result. Obviously, it relates to the miniature size of the expressed proteins, which were insufficiently long for efficient N-terminal processing, and it may be interesting to use our platform for further investigation of this phenomenon.

The use of a miniature pepA1 platform provided highly selective isolation and purification of target proteins from *E. coli* inclusion bodies, which was a valuable advantage as compared to constructs based on the SUMO platform. The high solubility of short L_6_KD-pepA1 fusions in the hydrophobic phase results in the high efficacy of the urea protein extraction method, which significantly facilitates SAPN purification and reduces its cost.

Nanoparticle size plays a significant role in drainage through the lymphatic system and interactions with antigen-presenting cells 2. The substitution of SUMO protein with pepA1 peptide led to a decrease in the SAPN diameter from 16 nm to approximately 10 nm and a switch from the fibers to the spherical micelles (Figure 4-6). Along with the increase in target-to-platform peptide size ratio, these changes can account for the higher immunogenicity of the pepA1-based SAPN as compared to the previous generation platform 6. In particular, we consistently reached a 100% seroconversion rate against target peptides in C57BL/6 mice after two-shot immunization (Figure 7), whereas only 50%-67% rates were observed for the SUMO-based SAPN under the similar immunization regimen 6. Moreover, against the target antigen PhtD19, the pepA1-based SAPN induced mean log_10_ antibody titer value ~ 4.7 (Figure 7) that is a stronger antigen-specific IgG response as compared to the SUMO-based nanoparticles (3.6 - 3.9) 6. Impressive results were also obtained for SAPN-OVA24. In an adjuvant-free regimen, SAPN-OVA24 induced an approximately five-fold higher antibody response against the target antigen as compared to the corresponding dose of the free OVA24 peptide administered with Freund's adjuvant (Figure 7). The high immunogenicity observed for both target antigens presented by the pepA1-based SAPN suggests the versatility of the platform (Figure 7), which is essential for vaccine development.

We believe that the most important advantage of the novel platform over its previous version is its substantially reduced relative immunogenicity of the adaptor peptide, which reflects the non-specific immunogenicity of the platform. In particular, pepA1-based SAPN predominantly induced an antigen-specific, rather than pepA1-specific IgG immune response, with the observed difference being statistically significant (p < 0.05, Figure 7). According to the results, the ratio of mean antibody titers to pepA1 versus target antigens in SAPN-PhtD19 and SAPN-OVA24 was approximately 20% and 7%, respectively (Figure 7). In contrast, for the previous version of SAPN, the immunogenicity of SUMO was comparable to or even slightly higher than that of the target antigen 6.

We assume that, among other things, the decreased relative immunogenicity of the pepA1 platform results from the shielding of the small pepA1 peptide with target surface antigens, which was not observed with the larger SUMO protein. However, the role of the shielding effect in the low immunogenicity of the pepA1-based platform as well as the potential contribution of the oligomerization capacity of pepA1 34 remains to be elucidated.

As mentioned above, the novel adaptor peptide pepA1 was designed on the basis of the human hormone Glp1. The substitution of approximately half the number of amino acid residues with their synonymous analogues enabled us to reduce the risk of an autoimmune cross-reactive response to Glp1 after administration of pepA1-based SAPN. Unfortunately, the low immunogenicity of pepA1 (Figure 7) hampered the evaluation of cross-reactivity between Glp1 and its derivative yet. We believe that due to the poor immunogenicity of the platform, the remnant similarity of pepA1 and Glp1 structures does not limit the application of SAPN for research and medical purposes. However, the real cross-reactivity between pepA1 and Glp1 as well as the possible ways to reduce it need to be explored.

Follicular helper T cells are essential for B-cell maturation and immunoglobulin production after immunization with thymus-dependent antigens 35. Earlier, similar SAPN preparations based on α‑helical peptide were shown to efficiently generate both B- and T-cellular responses 4. However, in our experiments, the induction of antigen-specific IgG in animals immunized with SAPN-OVA24 was not accompanied by a pronounced T cell reaction (Figure S3, Figure S4). Though this inconsistency can be related to the use of different antigens mediating distinguished features of the immune response, we believe that the optimization of the measurement protocol in the future will enable us to detect T cell responses induced by pepA1-based SAPN. To sum up, in many respects, the miniature pepA1-based platform surpasses the previous generation SUMO-based platform, and for this reason, it is more promising for future applications and can serve as a basis for the development of vaccines and other targeted medicines.

## Conclusions

In the current work, we have developed and experimentally characterized a miniature second-generation platform that provides efficient biosynthesis and self-assembly of SAPN displaying target antigens on their surface. In contrast to its ancestor, SUMO-based nanoparticles, pepA1-based SAPN induce a stronger antigen-specific antibody response in immunized animals and are characterized by significantly reduced self-immunogenicity of the platform. The flexibility of the system, which allows development of nanoparticles carrying different target molecules, along with their efficient production in *E. coli* cells, make pepA1-based SAPN a promising tool for various research and biomedicine purposes.

## Supplementary Material

Supplementary figures.

## Figures and Tables

**Table 1:** List of primers for plasmid construction

Primer №	Sequence (5' to 3')
1755	agcagatctaaaggtgacggttctttctctaccgaagttaccacctacctg
1756	gaagttaccacctacctggacggtaacgctgctcgtgactggatcgctttc
1757	agcctcgagttaggatcctttaccacgaaccaggaaagcgatccagtcacg
gs5rc	tatctcgagttaggatcctccaccaccagatctcttttccatggtatatctccttcttaaag
1734	ggtcatccagcggatagttaat
1971	tatctcgagttagaccttgatcttacgctcctccatgacgttggaactagtccactcagt
1972	ggaactagtccactcagtcaacttttcgaagttgatgatagatctcgatcctctacg
1727	tattagatctcatggtgaaggtacctttacca
619*	tatctcgagttaggatcctctgcctttcaccag

**Table 2:** List of primers for RT-PCR.

Gene	Primer	Sequence (5' to 3')
*Actb*	Forward	ctcctgagcgcaagtactctgtg
	Reverse	ctcctgagcgcaagtactctgtg
*Ifng*	Forward	tcaagtggcatagatgtggaagaa
	Reverse	tggctctgcaggattttcatg
*Il2*	Forward	acctctgcggcatgttctgg
	Reverse	agaaagtccaccacagttgctg
*Il4*	Forward	gccgatgatctctctcaagtgat
	Reverse	ggtctcaacccccagctagt

**Table 3:** Sequences of candidate peptides, derived from Glp1(Ala8Gly)^#^

Peptide	Sequence (5' to 3')
Glp1(Ala8Gly)	HGEGTFTSDVSSYLEGQAAKEFIAWLVKGR
pepA1	K*D*S*S*E*TT**D*N**RDW**F**R*K
pepA2	Y*Q*S*S**A*****A*S***W***S****
pepA3	F*S*A*A**A****D***G**W***S****

# amino acid residues not affected by mutagenesis are shown with asterisks

**Table 4:** Mass-spectrometry analysis of SAPN-proteins

Protein	Molecular weight, Da		
	Calculated*	Experimental	Delta
L_6_KD-pepA1-OVA24	7320.0	7480.3	160.3
L_6_KD-pepA1-gs5-PhtD19	7178.3	7339.6	161.3

* Calculation was performed for corresponding proteins lacking formylmethionine using Vector NTI software.

**Table 5:** The number of monomeric subunits in the composition of SAPN, according to the DLS data*

SAPN	Hydrodynamic radius, nm ± SD**	SAPN mass, kDa ± SD	Monomer mass, Da	Number of monomers
SAPN-OVA24	6.8 ± 2.8	296 ± 133	7320	**40 ± 18**
SAPN-PhtD19	5.1 ± 1.4	149 ± 47	7178.32	**21 ± 7**
	6.8 ± 2.6	296 ± 127		**41 ± 18**

*The measurement results of independently obtained specimens are presented. **Average radius of SAPN that formed the main peak in the intensity versus particle size distribution.

## References

[1] Gomes AC, Mohsen M, Bachmann MF (2017). Harnessing Nanoparticles for Immunomodulation and Vaccines. Vaccines (Basel).

[2] Roth GA, Picece V, Ou BS, Luo W, Pulendran B, Appel EA (2022). Designing spatial and temporal control of vaccine responses. Nat Rev Mater.

[3] Morales-Hernandez S, Ugidos-Damboriena N, Lopez-Sagaseta J (2022). Self-Assembling Protein Nanoparticles in the Design of Vaccines: 2022 Update. Vaccines (Basel).

[4] Wu Y, Norberg PK, Reap EA, Congdon KL, Fries CN, Kelly SH (2017). A Supramolecular Vaccine Platform Based on alpha-Helical Peptide Nanofibers. ACS Biomater Sci Eng.

[5] Koul B, Poonia AK, Yadav D, Jin JO (2021). Microbe-Mediated Biosynthesis of Nanoparticles: Applications and Future Prospects. Biomolecules.

[6] Gorbunov AA, Sannikova EP, Gubaidullin II, Serobyan GA, Gorbunova AY, Serkina AV (2022). Vaccine building 'kit': combining peptide bricks to elicit a desired immune response without adding an adjuvant. Nanomedicine (Lond).

[7] Vauthey S, Santoso S, Gong H, Watson N, Zhang S (2002). Molecular self-assembly of surfactant-like peptides to form nanotubes and nanovesicles. Proc Natl Acad Sci U S A.

[8] von Maltzahn G, Vauthey S, Santoso S, Zhang S (2003). Positively Charged Surfactant-like Peptides Self-assemble into Nanostructures. Langmuir.

[9] Zhao Q, Zhou B, Gao X, Xing L, Wang X, Lin Z (2017). A cleavable self-assembling tag strategy for preparing proteins and peptides with an authentic N-terminus. Biotechnol J.

[10] Zhou B, Xing L, Wu W, Zhang XE, Lin Z (2012). Small surfactant-like peptides can drive soluble proteins into active aggregates. Microb Cell Fact.

[11] Komolov AS, Sannikova EP, Gorbunov AA, Gubaidullin II, Plokhikh KS, Konstantinova GE (2024). Synthesis of biologically active proteins as L_6_KD-SUMO fusions forming inclusion bodies in Escherichia coli. Biotechnol Bioeng.

[12] Johnson ES, Schwienhorst I, Dohmen RJ, Blobel G (1997). The ubiquitin-like protein Smt3p is activated for conjugation to other proteins by an Aos1p/Uba2p heterodimer. EMBO J.

[13] Malakhov MP, Mattern MR, Malakhova OA, Drinker M, Weeks SD, Butt TR (2004). SUMO fusions and SUMO-specific protease for efficient expression and purification of proteins. J Struct Funct Genomics.

[14] Li SJ, Hochstrasser M (2003). The Ulp1 SUMO isopeptidase: distinct domains required for viability, nuclear envelope localization, and substrate specificity. J Cell Biol.

[15] Papastamatiou T, Routsias JG, Koutsoni O, Dotsika E, Tsakris A, Spoulou V (2018). Evaluation of Protective Efficacy of Selected Immunodominant B-Cell Epitopes within Virulent Surface Proteins of Streptococcus pneumoniae. Infect Immun.

[16] Kozlov DG, Sannikova EP, Klebanov FA, Cheperegin SE, Bulushova NV, Zalunin IA, Gracheva TS, Grachev SA, Yurin VL, Rykalina NV, Askerova EV, Yarotskii SV (2016). Polypeptide for reducing blood sugar levels based on the human glucagon-like peptide-1, recombinant E. coli producer strain and method for producing this polypeptide.

[17] Sannikova EP, Bulushova NV, Cheperegin SE, Zalunin IA, Klebanov FA, Gracheva TS (2019). Specific Activity of Recombinant Modified Human Glucagon-Like Peptide 1. Applied Biochemistry and Microbiology.

[18] Studier FW (2005). Protein production by auto-induction in high density shaking cultures. Protein Expr Purif.

[19] Grabski A, Mehler M, Drott D (2005). The Overnight Express Autoinduction System: High-density cell growth and protein expression while you sleep. Nature Methods.

[20] Laemmli UK (1970). Cleavage of structural proteins during the assembly of the head of bacteriophage T4. Nature.

[21] Peters GS, Zakharchenko OA, Konarev PV, Karmazikov YV, Smirnov MA, Zabelin AV (2019). The small-angle X-ray scattering beamline BioMUR at the Kurchatov synchrotron radiation source. Nuclear Instruments and Methods in Physics Research Section A: Accelerators, Spectrometers, Detectors and Associated Equipment.

[22] Peters GS, Gaponov YA, Konarev PV, Marchenkova MA, Ilina KB, Volkov VV (2022). Upgrade of the BioMUR beamline at the Kurchatov synchrotron radiation source for serial small-angle X-ray scattering experiments in solutions. Nuclear Instruments and Methods in Physics Research Section A: Accelerators, Spectrometers, Detectors and Associated Equipment.

[23] Hammersley AP (2016). FIT2D: a multi-purpose data reduction, analysis and visualization program. Journal of Applied Crystallography.

[24] Konarev PV, Volkov VV, Sokolova AV, Koch MHJ, Svergun DI (2003). PRIMUS: a Windows PC-based system for small-angle scattering data analysis. Journal of Applied Crystallography.

[25] Manalastas-Cantos K, Konarev PV, Hajizadeh NR, Kikhney AG, Petoukhov MV, Molodenskiy DS (2021). ATSAS 3.0: expanded functionality and new tools for small-angle scattering data analysis. J Appl Crystallogr.

[26] Kulvelis Y, Lebedev V, Yudina E, Shvidchenko A, Aleksenskii A, Vul A (2020). Structural Studies of Detonation Nanodiamonds with Grafted Metal Ions by Small-Angle Neutron Scattering. Journal of Surface Investigation: X-ray, Synchrotron and Neutron Techniques.

[27] Livak KJ, Schmittgen TD (2001). Analysis of relative gene expression data using real-time quantitative PCR and the 2(-Delta Delta C(T)) Method. Methods.

[28] Kallberg M, Wang H, Wang S, Peng J, Wang Z, Lu H (2012). Template-based protein structure modeling using the RaptorX web server. Nat Protoc.

[29] Riccione KA, He LZ, Fecci PE, Norberg PK, Suryadevara CM, Swartz A (2018). CD27 stimulation unveils the efficacy of linked class I/II peptide vaccines in poorly immunogenic tumors by orchestrating a coordinated CD4/CD8 T cell response. Oncoimmunology.

[30] Bogeholz LAK, Mercier E, Wintermeyer W, Rodnina MV (2021). Kinetic control of nascent protein biogenesis by peptide deformylase. Sci Rep.

[31] Ura T, Takeuchi M, Kawagoe T, Mizuki N, Okuda K, Shimada M (2022). Current Vaccine Platforms in Enhancing T-Cell Response. Vaccines (Basel).

[32] Romagnani S (1991). Type 1 T helper and type 2 T helper cells: functions, regulation and role in protection and disease. Int J Clin Lab Res.

[33] Zhao Q, Xu W, Xing L, Lin Z (2016). Recombinant production of medium- to large-sized peptides in Escherichia coli using a cleavable self-aggregating tag. Microb Cell Fact.

[34] Brichtová EP, Krupová M, Bouř P, Lindo V, Gomes dos Santos AL, Jackson SE (2023). Glucagon-like peptide 1 aggregates into low-molecular-weight oligomers off-pathway to fibrillation. Biophysical Journal.

[35] Craft JE (2012). Follicular helper T cells in immunity and systemic autoimmunity. Nat Rev Rheumatol.

