# Supplementary material for: A miniature low-immunogenic platform for the biosynthesis of self-assembling protein nanoparticles
Source: Nanotheranostics. 2025 Mar 3;9(1):67–81. doi: 10.7150/ntno.98946 (PMC11898719; doi:10.7150/ntno.98946)
Supplement: Supplementary file 1 — Supplementary figures. [file ntnov09p0067s1.pdf]

Supplementary materials

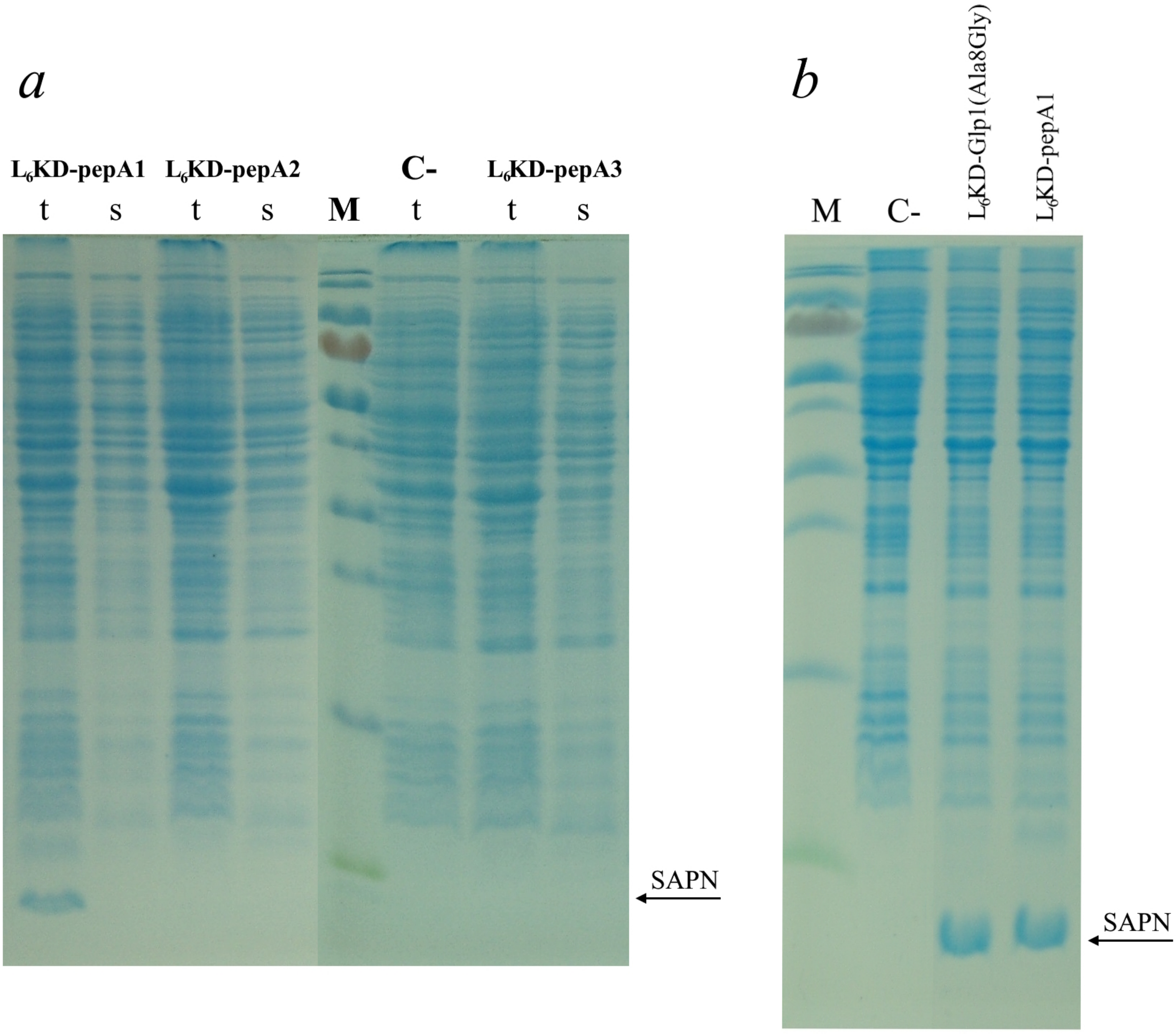

Figure S1. Comparison of expression level and solubility of fusion proteins L_6KD-pepA1, L_6KD-pepA2, L_6KD-pepA3 and L_6KD-Glp1(Ala8Gly) synthesized in *E. coli* BL21(DE3) cells. *a* - Total (t) and soluble (s) cellular fractions or *b* – total cellular fractions were analyzed. *Analysis was performed via 15% SDS-PAGE. Proportional amounts of samples were loaded into the wells. SAPN fusion proteins are indicated by arrows. C- negative control. M - molecular weight markers (BioRad 161-0373, MW 250, 150, 100, 75, 50, 37, 25, 20, 15, 10 kDa).*

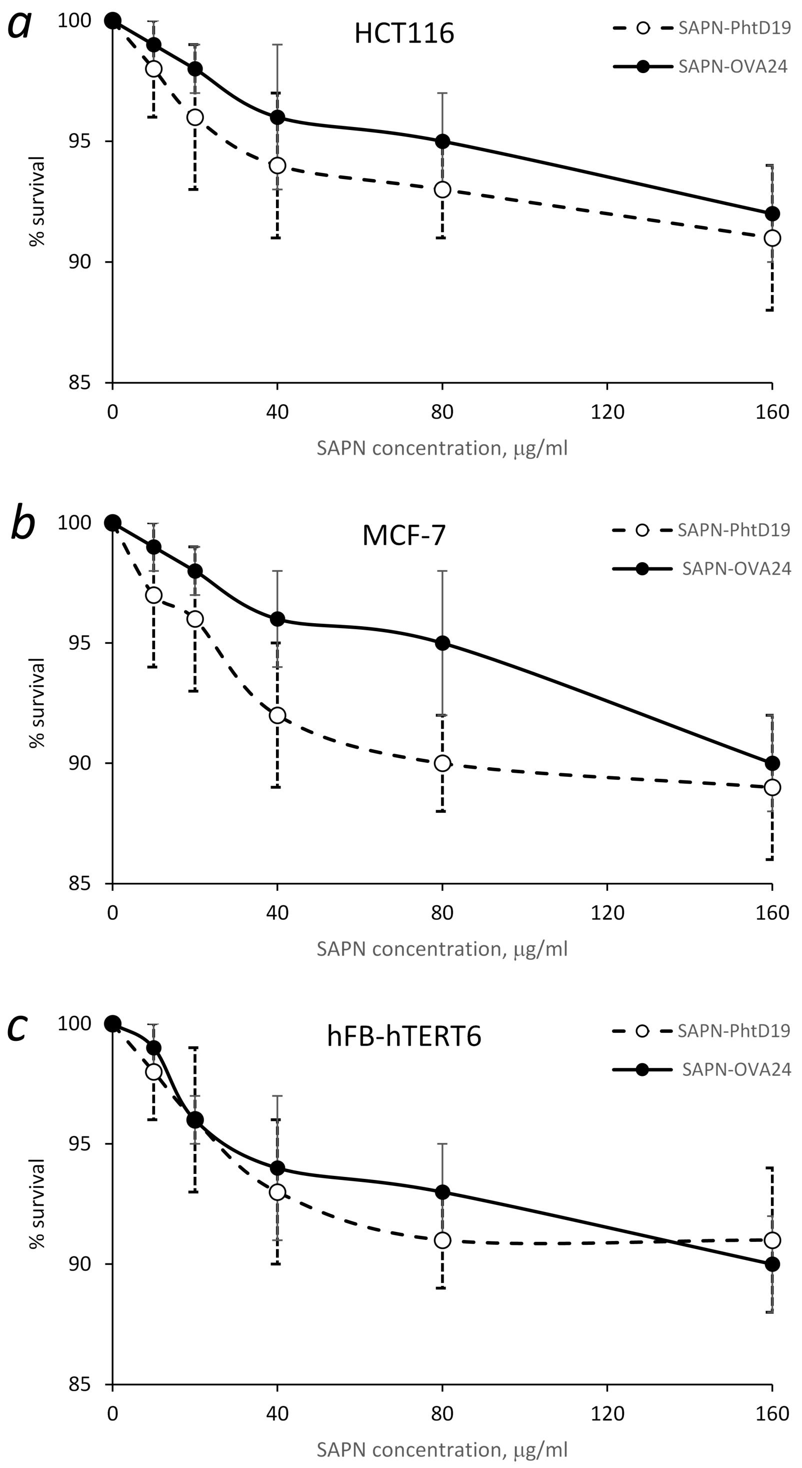

Figure S2. Viability of cells treated with SAPN fusion proteins. Indicated cell lines were treated with SAPN-PhtD19 or SAPN-OVA24 for 72 h at 37 ^{0}C, 5% CO_2 followed by MTT-assays. Data are mean $\pm$ SD of three measurements, each concentration tested in triplicate.

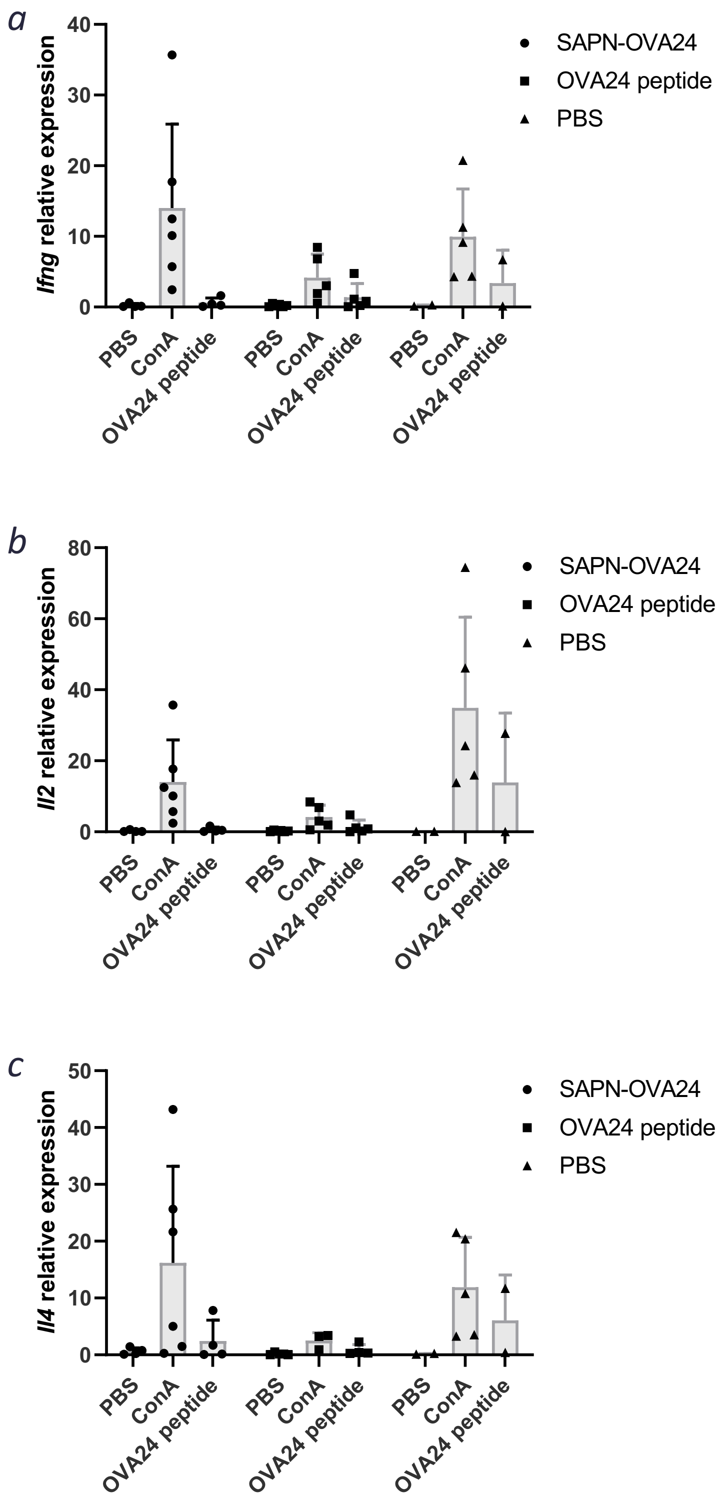

Figure S3. Relative cytokine mRNA expression in splenocytes from immunized mice. C57BL/6 mice were injected twice intraperitoneally with 50 µg SAPN-OVA24, the equivalent dose of the OVA24 peptide or PBS. On day 28 splenocytes were isolated and stimulated with ConA, SAPN-OVA24 or vehicle for 6 h at 37 ^{0}C, 5% CO_2. *Ifng* (a), *Il2* (b) and *Il4* (c) mRNA expression was measured by RT-PCR. Relative expression values were referred to *Actb* expression. Data are the mean ± SD of 2 measurements (n =2-6 mice per group).

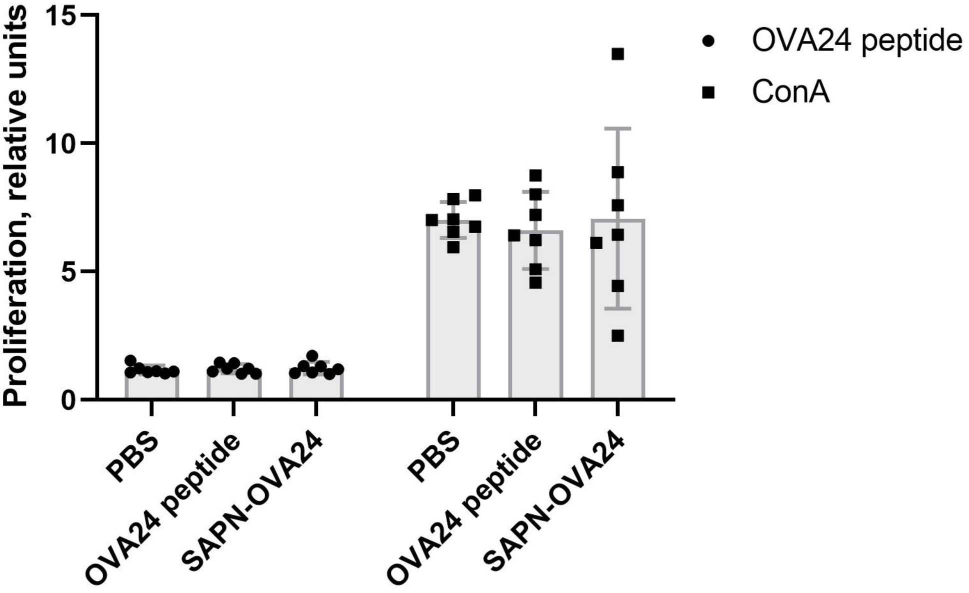

Figure S4. Proliferation of splenocytes from immunized mice. C57BL/6 mice were injected twice intraperitoneally with 50 µg SAPN-OVA24, the equivalent dose of the OVA24 peptide or PBS. On day 28 splenocytes were isolated and stimulated with ConA or SAPN-OVA24 for 45 h at 37 ^{0}C, 5% CO_2 followed by MTT-assay. Cell proliferation was calculated by dividing absorbance in wells with treated cells to that of untreated cells. Data are the mean ± SD of 4 measurements (n =7 mice per group).
